# Case report: Watch-and-wait strategy in resectable esophageal cancer following neoadjuvant chemoimmunotherapy: a case series

**DOI:** 10.3389/fimmu.2024.1502206

**Published:** 2025-01-06

**Authors:** Lingyu Tan, Guozhen Yang, Chufeng Zeng, Xu Zhang

**Affiliations:** ^1^ Department of Thoracic Oncology, Sun Yat-sen University Cancer Center, Guangzhou, China; ^2^ State Key Laboratory of Oncology in South China, Collaborative Innovation Center for Cancer Medicine, Sun Yat-sen University Cancer Center, Guangzhou, China; ^3^ Guangdong Esophageal Cancer Institute, Guangzhou, China

**Keywords:** neoadjuvant chemoimmunotherapy, watch and wait, esophageal cancer, clinical complete response, PD-1 blockade

## Abstract

Neoadjuvant chemoimmunotherapy (NCIT) has improved pathological complete response and conferred survival benefits in patients with locally advanced esophageal cancer. However, surgical complications unrelated to the tumor continue to detract from patient outcomes. While the “watch-and-wait” strategy has been implemented in clinical complete responders following neoadjuvant therapy for rectal cancer, there is a lack of evidence supporting its practicability in esophageal cancer after NCIT. This pilot case series involves six clinical complete responders who deferred surgery under close surveillance after three or four cycles of neoadjuvant camrelizumab plus chemotherapy and who subsequently received camrelizumab as maintenance treatment. The primary observation measure of the series is event-free survival (EFS). Routine follow-up examinations included endoscopy, biopsy, contrast-enhanced computed tomography, and ultrasonography every 3–6 months. For patients who experienced local recurrence without metastasis, the salvage operation was the priority recommendation. As of September 5, 2024, the average follow-up duration was 124.4 weeks, with the average EFS reaching 134.7 weeks. No deaths or distant metastases were observed. Our findings suggest that responders to NCIT may be spared from esophagectomy. On the prerequisite of sufficient tumor regression during neoadjuvant cycles, immunotherapy may facilitate the continued eradication of residual disease in this series.

## Introduction

Esophageal cancer remains one of the most lethal malignancies globally (1), with a significant proportion of cases diagnosed at a locally advanced stage, predisposing patients to a high risk of recurrence and metastasis following surgery alone. Immune checkpoint inhibitors (ICIs) have demonstrated efficacy in both advanced and resectable esophageal cancer (2–6). The rise in the number of complete responders questions the absolute indispensability of surgery in certain cases. While minimally invasive surgical techniques have been developed to reduce the morbidity associated with esophagectomy, their impact remains insufficient to fully mitigate the challenges of this complex procedure (7). Owing to the unique anatomical and functional characteristics of the esophagus, postoperative complications (commonly pneumonia, anastomotic leakage, and gastrointestinal dysfunction) continue to greatly plague the patient’s quality of life (8). In this context, a “watch-and-wait” (W&W) approach has gained attention, leveraging the high rates of pathological complete response (pCR) and the growing preference for preserving the organs. While some studies have suggested that esophagectomy might not confer long-term benefits in clinical complete responders after neoadjuvant radiochemotherapy (NCRT) (9), others, particularly a large-scale retrospective study, have reported increased postoperative morbidity and reduced survival associated with delayed surgery (10). These conflicting findings underscore the uncertainty regarding the feasibility and safety of the W&W strategy in esophageal cancer. Moreover, there is limited evidence addressing whether NCIT might offer a more compatible alternative to classical NCRT in the context of W&W.

In this study, we report on six complete responders after NCIT. Owing to their refusal to surgery, ICIs were administered with the expectation of maintaining disease control. As of September 5, 2024, the average follow-up duration was 124.4 weeks.

## Case presentation

We evaluated six patients diagnosed with locally advanced thoracic esophageal squamous cell carcinoma (ESCC). Baseline assessments were performed using endoscopic ultrasonography (EUS, or normal endoscopy with esophageal narrowness), contrast-enhanced computed tomography (CT) or magnetic resonance imaging (MRI), and ultrasonography of the cervical lymph nodes. Each patient received three or four three-weekly neoadjuvant treatments with camrelizumab (200 mg m^−2^ day^−1^; Jiangsu Hengrui Pharmaceuticals, Shanghai, China) and nab-paclitaxel (260 mg m^−2^ day^−1^; CSPC Pharmaceutical, Shijiazhuang, China) on day 1, in combination with either S-1 (60 mg m^−2^ day^−1^, four cycles; Qilu Pharmaceutical, Shanghai, China) or capecitabine (1,250 mg m^−2^ day^−1^, three cycles; Jiangsu Hengrui Pharmaceuticals, Shanghai, China) from day 1 to day 14. Treatment response was evaluated after each cycle as previously described for the baseline assessments. Clinical complete response (cCR) was defined as the complete regression of both the primary tumor and the lymph nodes. The researchers identified the response through medical imaging (the same as the baseline) and EUS with biopsies after the second and the final administration of NCIT. Recovery of mucous integrity and the muscle layer’s continuity under EUS was considered complete regression. Each cCR status confirmed by endoscopy required biopsies, which included at least four bite-on-bite biopsies of the primary tumor. Cases demonstrating complete response on imaging but with suspected histopathological results (e.g., dysplasia) were classified as near-cCR.

Following completion of neoadjuvant therapy, all six patients received maintenance therapy with camrelizumab alone or in combination with S-1, having opted to forgo surgical intervention. Follow-up evaluations were conducted every 3 months through outpatient visits or telephone consultations to assess the survival status. Tumor recurrence and metastasis were monitored using the aforementioned imaging modalities, supplemented as needed with ^18^F-fluorodeoxyglucose positron emission tomography CT (^18^F-FDG PET/CT). In cases where the initial biopsy revealed indeterminate lesions (e.g., dysplasia or precancerous lesions), during the follow-up phase, EUS and a second biopsy will be conducted 3 months later. Event-free survival (EFS) was designated as the study outcome and was defined as the interval from the initiation of treatment to the occurrence of any of the following events: death from any cause, the first disease progression, or tumor metastasis.

**Figure 1:**
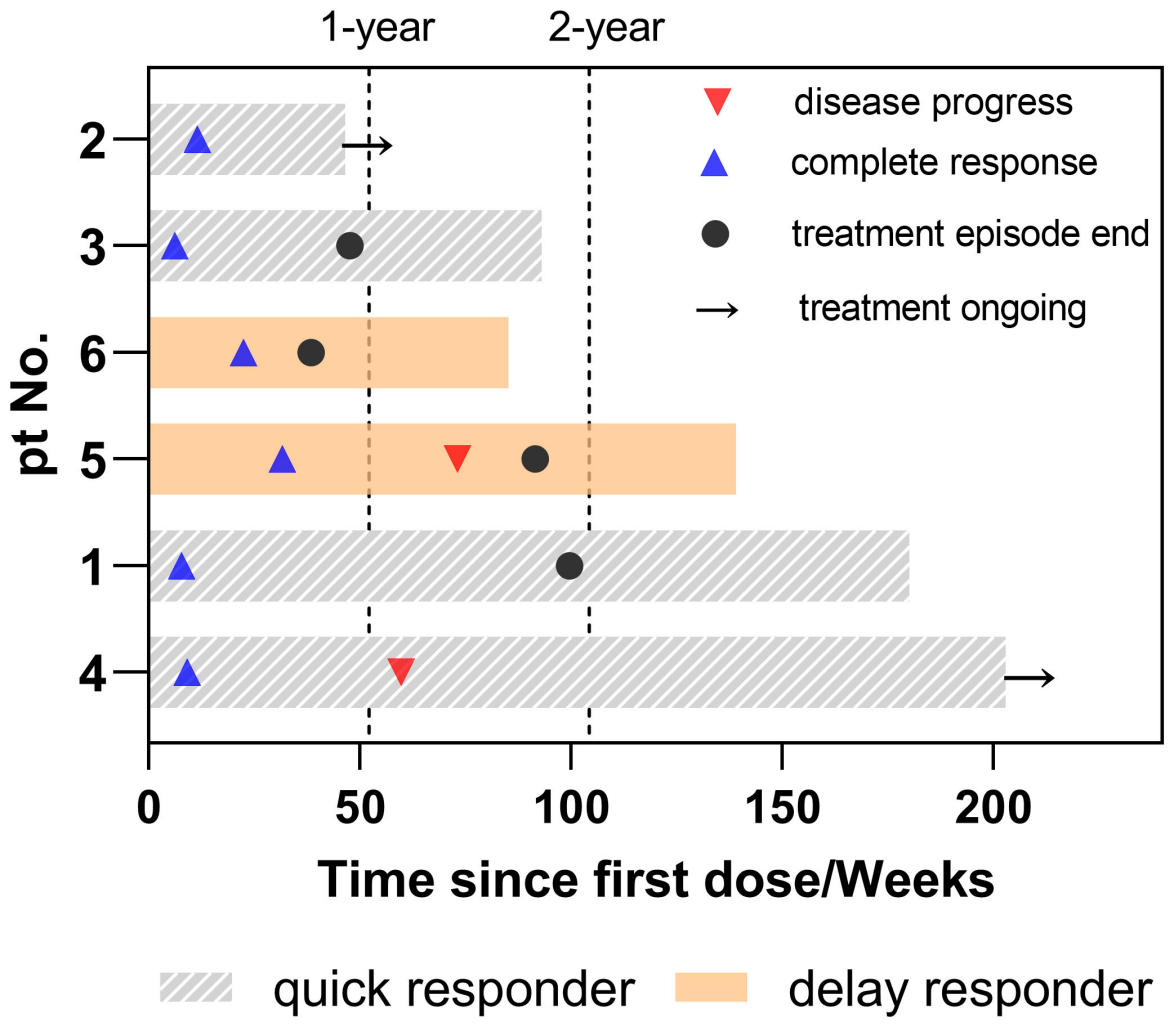
Duration of treatment in patients. Timeline of systematic treatment, including the duration of neoadjuvant and maintenance therapy. Disease progress refers to the time from the first dose to the first recurrence or metastasis. Complete response indicates the time from the first dose to the clinical complete response. Treatment episode end is the duration of neoadjuvant and maintenance therapy. Treatment ongoing indicates continuous maintenance treatment up to the endpoint of the study.

**Figure 2:**
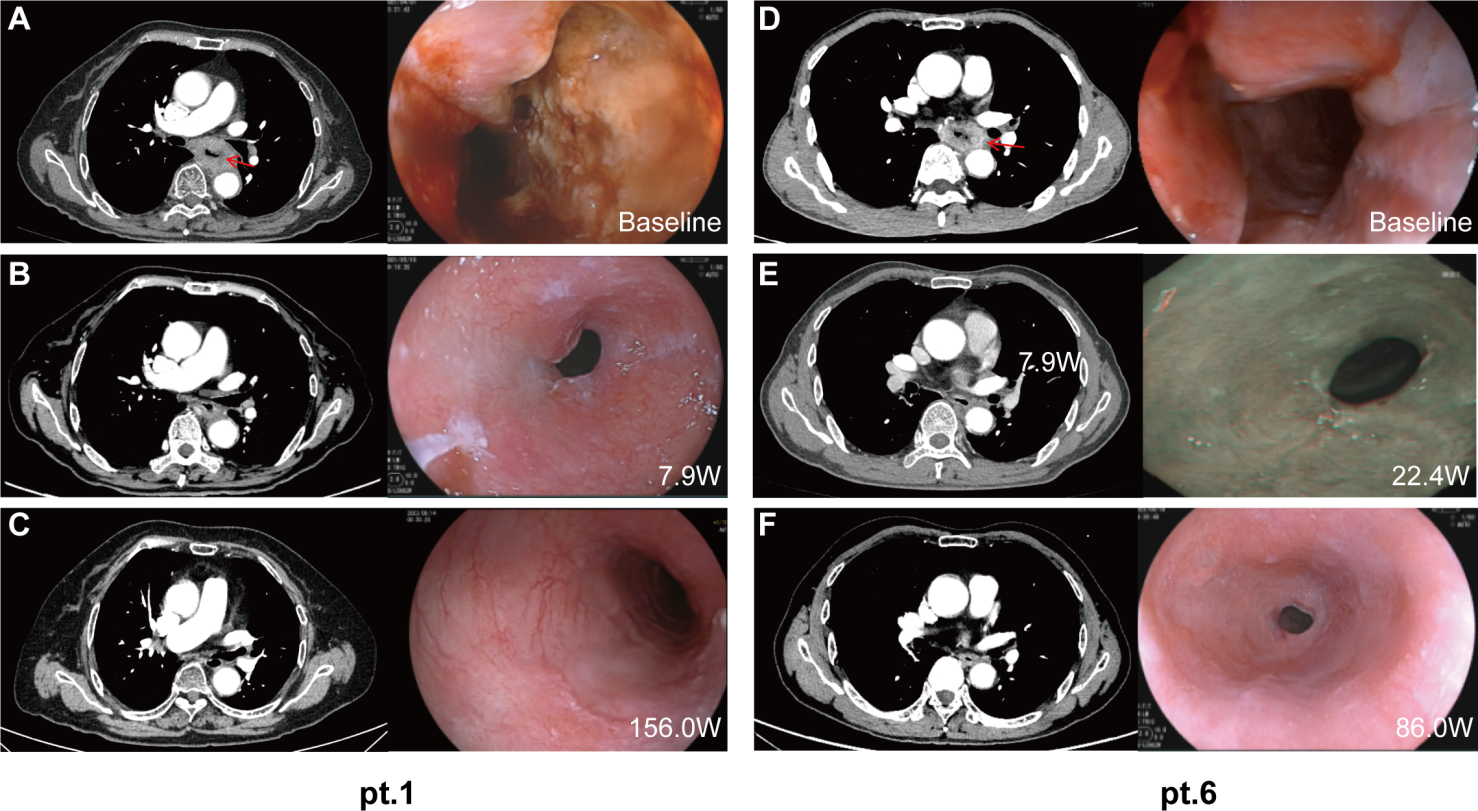
Radiology and endoscopic images of the primary tumor during treatment. Representative computed tomography and endoscopy images of patient 1 (quick responder) and patient 6 (delayed responder) at baseline **(A, D)**, at the time of clinical complete response **(B, E)**, and at the most recent follow-up **(C, F)**. *W*, weeks from the first dose.

The case summary of the patient characteristics and treatment process is shown in **Table 1**. None of the participants discontinued or prematurely suspended their treatment. Based on the initial response time to NCIT, patients were retrospectively categorized into two groups: quick responders and delayed responders. Representative radiological and endoscopy images of tumor regression are shown in **Figure 1**. Quick responders were defined as those achieving cCR within the scheduled neoadjuvant cycles, while delayed responders achieved cCR thereafter. The median time to cCR for the cohorts was 9 weeks (range = 8–32 weeks), and the median duration of maintenance treatment was 79.3 weeks (range = 22.6–189.1 weeks) (**Figure 2** shows the treatment timelines of the six patients). During the maintenance phase, two patients received camrelizumab monotherapy, while the remaining patients were treated with the camrelizumab and S-1 combination.

**Table 1:** Case summary.

Group		Gender	Age	Location	Grade	cTNM	Neoadjuvant chemotherapy*	Cycles (cCR/all) ^#^	Maintenance treatment		EFS/weeks	OS/weeks
									Regimen	Duration/weeks		
Q	1	F	81	Middle	Moderate	III (T3N2M0)	Nab-paclitaxel, capecitabine (C1), S-1 (C2–C4)	2/4	Camrelizumab, S-1	82	80	180
	2	M	68	Low	Poor	III (T2N2M0)	Nab-paclitaxel, S-1	4/4	Camrelizumab, S-1	35	47	47
	3	M	70	Low	Moderate	III (T3N2M0)	Nab-paclitaxel, capecitabine	2/3	Camrelizumab, S-1	24	93	93
	4	F	73	Middle	Poor	III (T3N2M0)	Nab-paclitaxel, S-1	3/4	Camrelizumab	189	60	202
D	5	F	75	Middle	Moderate	III (T3N2M0)	Nab-paclitaxel, S-1	9/4	Camrelizumab, S-1	79	73	139
	6	M	64	Low	Poor	III (T3N2M0)	Nab-paclitaxel, S-1	7/4	Camrelizumab	23	85	85

*All patients received immunotherapy of camrelizumab. #cCR/all: Total treatment cycles prior to cCR (including maintenance treatment) / cycles of neoadjuvant treatment; D: delayed response; Q: quick response; F: Female; M: Male; C: cycle; S-1: Tegafur Gimeracil Oteracil Potassium Capsule; OS: overall survival; EFS: event-free survival; Pt: patient.

Two individuals from the different groups experienced local recurrence. Patient 5 (a delayed responder) was diagnosed with early cancer during the 41st week of his maintenance phase via EUS. Concurrent biopsy findings indicated atypical hyperplasia, which could not definitively exclude the possibility of more severe lesions. A subsequent EUS without biopsy, which was conducted 4 months later, revealed no disease progression and was classified as type B1 using narrow-band imaging. This patient continued maintenance therapy for a total of 79.3 weeks until the endpoint of the study. Patient 4 (a quick responder) was diagnosed with uTis (tumor *in situ*) in the 51st week after achieving cCR. Despite this progression, the patient maintained surgery refusal and continued camrelizumab monotherapy for 189.1 weeks as of the latest follow-up, without dysphagia or any distant metastasis reported. Although postponement of surgery could result in compromised survival, both patients persisted in refusing surgical interventions. The rationale for this decision included their advanced age (both patients were over 75 years old at the time of diagnosis or recurrence), the absence of significant symptoms such as weight loss or dysphagia, and the lack of evidence of distant metastases on imaging. Both patients were undergoing strict follow-up evaluations every 3 months, and surgery was strongly recommended should symptoms worsen or disease progression be detected on imaging. ICIs would be continued unless intolerable adverse events occur or the patient elects to discontinue therapy.

No treatment discontinuation or dosage reduction due to adverse events occurred in this cohort. The average EFS was 134.7 weeks [95% confidence interval (CI) = 85.7–183.6 weeks], with the median EFS not yet reached. No deaths or distant metastases have been observed to date.

## Discussion

NCRT followed by surgery is the recommended treatment for locally advanced esophageal cancer (11, 12). It not only results in significant pCR rates but also confers substantial long-term survival benefits compared with surgery alone. However, surgery, which is considered as the cornerstone of comprehensive treatment, is inherently traumatic and commonly results in a higher risk of perioperative morbidity and mortality unrelated to the malignancy itself. These risks, coupled with the impact on long-term quality of life, have led to a growing preference among patients for the W&W strategy following cCR to neoadjuvant therapy. The notable pCR rates observed postoperatively, along with their potential to transform into survival benefits, provide a theoretical foundation for investigating the feasibility of the W&W strategy.

The W&W strategy has been extensively practiced in patients with rectal cancer who respond significantly to neoadjuvant therapy. For rectal cancer, a pCR rate of 15%–27% has been reported following NCRT, with corresponding improvements in long-term outcomes (13). The three-tier cCR diagnostic criteria pioneered by Memorial Sloan Kettering Cancer Center (MSKCC), which integrate rectal palpation, MRI, endoscopy, and biopsy, have been proven effective in minimizing misdiagnoses arising from discrepancies between clinical and pathological assessments (14). Based on this framework, W&W has been established as an optional strategy for patients with rectal cancer who achieve cCR, comprising intensive follow-up and salvage surgery in the event of local tumor regrowth. In 2022, MSKCC published findings on patients who achieved cCR after neoadjuvant ICIs were exempted from surgery, with progression-free survival exceeding 25 months (15). While the impact of this approach on distant metastasis remains debated, analyses of the overall survival (OS) indicated no significant difference in the outcomes between patients managed with the W&W strategy and those undergoing conventional surgery (16, 17). Notably, local recurrence occurred in approximately 15%–25% of patients adopting W&W, predominantly within 2–3 years of achieving cCR. Local recurrence occurred in approximately 15% to 25% of patients, predominantly within two to three years after achieving cCR. Local recurrence occurred in approximately 15% to 25% of patients, predominantly within two to three years after achieving cCR. Importantly, salvage surgery was feasible in 80% to 90% of these cases (18–21). However, it must be emphasized that the W&W strategy may be hazardous in the more aggressive tumors. In a retrospective study on liver cancer, the W&W group showed similar 3-year OS, but significantly lower PFS rates compared with the pCR group. Moreover, 35.3% (18/51) of recurred patients underwent curative treatments (22).

Despite ongoing advancements, the role of organ preservation management for esophageal cancer after NCRT remains contentious, particularly with respect to long-term survival and the incidence of perioperative complications (23, 24). The preliminary findings of the “Surgery *versus* Active Surveillance for Oesophageal Cancer” (preSANO) study proposed a promising multimodal approach to assess cCR (25). However, the recently updated results from the SANO trial revealed that only 35% of patients sustained cCR at 2 years, with the local recurrence rates exceeding 40%, consistent with prior retrospective analyses (26). These findings suggest that enhanced diagnostic accuracy in cCR assessment has not translated into a reduction in the recurrence rates. Beyond monitoring and detection, the role of interventions such as maintenance therapy warrants further exploration.

The advent of ICIs has led to a gradual shift toward neoadjuvant systemic therapies, but it remains unclear whether the patterns of tumor regression and their durability differ between NCRT and NCIT. In this study, endoscopy, CT, ultrasound of superficial lymph nodes, and multiple punch biopsies were used to evaluate residual lesions. Two patients who had sustained cCR for at least 10 months experienced local recurrences, including one case that progressed incrementally from a precancerous lesion to T2. We admit that this series had very limited statistical power given the small sample. The predictive value of response time is still uncertain. It is intriguing to further explore the differences in dynamic microenvironment changes between these two types of patients. That aside, this raises critical questions about the origins of recurrence: does it stem from residual disease or from malignant transformation within seemingly normalized mucosal tissue? Once cCR is conceptualized as a dynamic phase of the disease rather than a stable state, continued antitumor therapy may be necessary to sustain this phase, a concept not emphasized in the SANO study. In rectal cancer, local recurrence occurs in approximately 15%–25% of patients undergoing the W&W strategy (18–21). However, esophageal cancer presents distinct challenges due to its predisposition for early metastasis. Even patients undergoing surgery after neoadjuvant therapy face substantial risks of recurrence and metastasis. These risks are even greater in the absence of surgery. Accordingly, maintenance therapy following cCR may be indispensable for improving outcomes.

The efficacy of systemic treatment modalities following surgery for esophageal cancer was ambiguous before the introduction of ICIs. The CheckMate 577 trial provided robust evidence supporting the use of nivolumab following NCRT in esophageal cancer, demonstrating their efficacy in the postoperative setting (27), Furthermore, ICIs have shown promise in the adjuvant treatment of lung cancer and other solid tumors, underscoring their broad therapeutic potential (28–31). Emerging evidence also suggests benefits associated with the “sandwich regimen,” which integrates immunotherapy into perioperative care. Recent findings from the AEGEAN and NADIM II trials have demonstrated significant improvements in the 2-year survival and pCR rates with perioperative immunotherapy compared with chemotherapy alone (31, 32). ICIs are efficacious in addressing both local recurrence and distant micrometastases, which could persist after neoadjuvant therapy. In this study, two patients initially staged as ycT1N0M0 following neoadjuvant therapy exhibited sustained tumor regression and ultimately achieved cCR after several cycles of maintenance therapy (without intravenous chemotherapy). On the precondition of the major tumor regression after neoadjuvant cycles, immune maintenance therapy ultimately brought about complete response (within an additional three and five cycles). Regardless of the initial response, both subgroups experienced one recurrence during the maintenance phase. The effectiveness of maintenance treatment to control recurrence and metastasis still needs to be confirmed through survival benefits in randomized controlled studies. Due to the lack of prospective research, caution should be exercised regarding the conditions reported in this study. The timing of drug discontinuation is another concern in clinical events. Prognostic analyses suggest that the critical period for disease progression in nonsurgical populations is approximately 2 years (33). Accordingly, the patients in this cohort underwent maintenance treatment for a median duration of 79.3 weeks, with two patients continuing oral maintenance therapy to date. Prospective evidence from the CheckMate 153 study highlights the potential for sustained benefits from ICIs with treatment durations of at least 2 years (34). In addition, pooled long-term data on nivolumab from four clinical trials revealed a survival risk plateau beginning at 3 years, with benefits persisting through the 4-year endpoint (35). However, the precise parameters of this “tailing effect” remain to be elucidated and warrant further investigation.

Given the strong preference for organ preservation among patients achieving cCR, as well as the relatively modest pCR rates of 25%–50% associated with NCIT in advanced esophageal cancer (36–38), rigorous evaluation of cCR is critical to avoid "both" overtreatment and unexpected early recurrences. Previous studies indicated that approximately 30% of patients who achieve cCR also achieved pCR (39, 40). However, a meta-analysis evaluating the diagnostic accuracy of common modalities, including CT, EUS, MRI, and PET/CT, for cCR assessment demonstrated that none of these techniques consistently met diagnostic expectations (41). Endoscopic evaluations are particularly challenging, as post-radiotherapy fibrosis can obscure mucosal lesions, even when combined with ultrasound. Moreover, biopsies may not be feasible in cases of intact mucosa, further contributing to the high rates of missed diagnoses. Although multimodal examination is highly conservative in determining CCR, the specificity of any individual examination in monitoring recurrence is insufficient. Overall, the current methods for the evaluation of cCR following neoadjuvant treatment remain inadequate. At the level of molecular pathology, liquid biopsy to detect minimal residual disease (MRD) might be a promising approach. A prospective study used dynamic circulating tumor deoxyribonucleic acid (ctDNA) to predict outcomes in non-small cell lung cancer. Researchers classified patients into the rapid response and delayed response groups based on the ctDNA zeroing trend during NCRT and found that quick responders had better PFS (42). The results from the IMpower150 study showed that MRD had stronger prognostic effectiveness than radiographic imaging and that optimized risk stratification can help guide earlier clinical interventions (43). However, the impact of active intervention on the OS of patients with esophageal cancer is still unclear. In the real world, the increased burden of follow-up may significantly reduce the compliance of the W&W group. Ultimately, the value of MRD is pending further investigations.

Recently, gradual tumor regression with eventual restoration of the mucosal cavity has been identified as the predominant pattern of tumor regression (43.5%) among responders to ICIs, whereas stochastic regression is more commonly observed in those responding to chemotherapy or ICIs (51.4%) (44, 45). Although the nidus in the mucosal layer disappears, residual disease may persist in the muscular or deeper layers, which could partly explain the reduced accuracy of EUS in patients who have undergone NCRT. In this study, multiple endoscopic biopsies were performed, supplemented with CT, MRI, or PET/CT in four patients, as well as superficial lymph node ultrasound, to enhance the precision of clinical restaging after NCIT. Based on prior evidence and our findings, the specific regression patterns associated with ICIs may help in the identification of patients with a sustained response after achieving cCR. Further large-scale prospective studies are necessary to validate the utility of integrating pathological regression patterns with medical imaging in the assessment of cCR.

This study has several limitations. Firstly, it is a small-sample retrospective cohort, and all patients strongly preferred organ preservation. While salvage surgery was discussed as a treatment option, it was not pursued in any of the cases. In addition, due to the lack of a prospective protocol, this study did not evaluate biomarkers or immune profiles, which could have provided deeper insights into the mechanisms of response and resistance.

## Conclusion

We presented a small-scale case series of six patients who achieved cCR and refused surgery. The median - average follow-up time was 124.4 weeks, with EFS reaching 134.7 weeks as of September 5, 2024. Our findings suggest the potential feasibility of sparing complete responders from esophagectomy. Notably, sustained regression was observed in patients with delayed response during maintenance therapy, underscoring the possible importance of continued treatment after achieving cCR. The initial complete response duration and the tumor regression pattern may be potential indicators to predict outcomes. This study provides preliminary evidence supporting the feasibility of the W&W strategy in complete responders to NCIT in esophageal cancer. Future prospective trials are needed to establish the non-inferiority of this approach and to offer a new alternative for the elderly or those patients who are intolerant to surgery.

## Data Availability

The raw data supporting the conclusions of this article will be made available by the authors, without undue reservation.

## References

[1] Siegel RA-O Giaquinto AA-O Jemal A. Cancer statistics. CA Cancer J Clin. (2024) 74(1):12–49. doi: 10.3322/caac.21820 38230766

[2] Doki Y Ajani JA Kato K Xu J Wyrwicz L Motoyama S. Nivolumab combination therapy in advanced esophageal squamous-cell carcinoma. N Engl J Med. (2022) 386:449–62. doi: 10.1056/NEJMoa2111380 35108470

[3] Lu Z Wang J Shu Y Liu L Kong L Yang L. Sintilimab versus placebo in combination with chemotherapy as first line treatment for locally advanced or metastatic oesophageal squamous cell carcinoma (ORIENT-15): multicentre, randomised, double blind, phase 3 trial. BMJ. (2022) 377:e068714. doi: 10.1136/bmj-2021-068714 35440464 PMC9016493

[4] Wang ZX Cui C Yao J Zhang Y Li M Feng J. Toripalimab plus chemotherapy in treatment-naive, advanced esophageal squamous cell carcinoma (JUPITER-06): A multi-center phase 3 trial. Cancer Cell. (2022) 40:277–88.e3. doi: 10.1097/SLA.0000000000005798 35245446

[5] Yan X Duan H Ni Y Zhou Y Wang X Qi H. Tislelizumab combined with chemotherapy as neoadjuvant therapy for surgically resectable esophageal cancer: A prospective, single-arm, phase II study (TD-NICE). Int J Surg. (2022) 103:106680. doi: 10.1016/j.ijsu.2022.106680 35595021

[6] Qin J Xue L Hao A Guo X Jiang T Ni Y. Neoadjuvant chemotherapy with or without camrelizumab in resectable esophageal squamous cell carcinoma: the randomized phase 3 ESCORT-NEO/NCCES01 trial. Nat Med. (2024) 30(9):2549–2557. doi: 10.1038/s41591-024-03064-w 38956195 PMC11405280

[7] Mariette C Markar SR Dabakuyo-Yonli TS Meunier B Pezet D Collet D. Hybrid minimally invasive esophagectomy for esophageal cancer. N Engl J Med. (2019) 380:152–62. doi: 10.1056/NEJMoa1805101 30625052

[8] Derogar M Orsini N Sadr-Azodi O Lagergren P. Influence of major postoperative complications on health-related quality of life among long-term survivors of esophageal cancer surgery. J Clin Oncol. (2012) 30:1615–9. doi: 10.1200/JCO.2011.40.3568 22473157

[9] van der Wilk BJ Noordman BJ Neijenhuis LKA Nieboer D Nieuwenhuijzen GAP Sosef MN. Active surveillance versus immediate surgery in clinically complete responders after neoadjuvant chemoradiotherapy for esophageal cancer: A multicenter propensity matched study. Ann Surg. (2021) 274:1009–16. doi: 10.1097/SLA.0000000000003636 31592898

[10] Boerner T Harrington C Tan KS Adusumilli PS Bains MS Bott MJ. Waiting to operate: the risk of salvage esophagectomy. Ann Surg. (2023) 781(5):781–8. doi: 10.1097/SLA.0000000000005798 PMC10354214 36727949

[11] Yang H Liu H Chen Y Zhu C Fang W Yu Z. Long-term efficacy of neoadjuvant chemoradiotherapy plus surgery for the treatment of locally advanced esophageal squamous cell carcinoma: the NEOCRTEC5010 randomized clinical trial. JAMA Surg. (2021) 156:721–9. doi: 10.1001/jamasurg.2021.2373 PMC8223138 34160577

[12] Eyck BM van Lanschot JJB Hulshof M van der Wilk BJ Shapiro J van Hagen P. Ten-year outcome of neoadjuvant chemoradiotherapy plus surgery for esophageal cancer: the randomized controlled CROSS trial. J Clin Oncol. (2021) 39:1995–2004. doi: 10.1200/JCO.20.03614 33891478

[13] Maas M Nelemans PJ Valentini V Das P Rodel C Kuo LJ. Long-term outcome in patients with a pathological complete response after chemoradiation for rectal cancer: a pooled analysis of individual patient data. Lancet Oncol. (2010) 11:835–44. doi: 10.1016/S1470-2045(10)70172-8 20692872

[14] Smith JJ Chow OS Gollub MJ Nash GM Temple LK Weiser MR. Organ Preservation in Rectal Adenocarcinoma: a phase II randomized controlled trial evaluating 3-year disease-free survival in patients with locally advanced rectal cancer treated with chemoradiation plus induction or consolidation chemotherapy, and total mesorectal excision or nonoperative management. BMC Cancer. (2015) 15:767. doi: 10.1186/s12885-015-1632-z 26497495 PMC4619249

[15] Cercek A Lumish M Sinopoli J Weiss J Shia J Lamendola-Essel M. PD-1 blockade in mismatch repair-deficient, locally advanced rectal cancer. N Engl J Med. (2022) 386:2363–76. doi: 10.1056/NEJMoa2201445 PMC9492301 35660797

[16] Habr-Gama A Perez RO Proscurshim I Campos FG Nadalin W Kiss D. Patterns of failure and survival for nonoperative treatment of stage c0 distal rectal cancer following neoadjuvant chemoradiation therapy. J Gastrointest Surg. (2006) 10:1319–28; discussion 28-9. doi: 10.1016/j.gassur.2006.09.005 17175450

[17] Habr-Gama A Perez RO Nadalin W Sabbaga J Ribeiro U Jr. Silva ESAH Jr.. Operative versus nonoperative treatment for stage 0 distal rectal cancer following chemoradiation therapy: long-term results. Ann Surg. (2004) 240:711–7; discussion 7-8. doi: 10.1097/01.sla.0000141194.27992.32 15383798 PMC1356472

[18] Smith JJ Strombom P Chow OS Roxburgh CS Lynn P Eaton A. Assessment of a watch-and-wait strategy for rectal cancer in patients with a complete response after neoadjuvant therapy. JAMA Oncol. (2019) 5:e185896. doi: 10.1001/jamaoncol.2018.5896 30629084 PMC6459120

[19] Dattani M Heald RJ Goussous G Broadhurst J Sao Juliao GP Habr-Gama A. Oncological and survival outcomes in watch and wait patients with a clinical complete response after neoadjuvant chemoradiotherapy for rectal cancer A systematic review and pooled analysis. Ann OF Surg. (2018) 268:955–67. doi: 10.1097/SLA.0000000000002761 29746338

[20] Dossa F Chesney TR Acuna SA Baxter NN. A watch-and-wait approach for locally advanced rectal cancer after a clinical complete response following neoadjuvant chemoradiation: a systematic review and meta-analysis. Lancet Gastroenterol HEPATOLOGY. (2017) 2:501–13. doi: 10.1016/S2468-1253(17)30074-2 28479372

[21] van der Valk MJM Hilling DE Bastiaannet E Kranenbarg EM-K Beets GL Figueiredo NL. Long-term outcomes of clinical complete responders after neoadjuvant treatment for rectal cancer in the International Watch & Wait Database (IWWD): an international multicentre registry study. LANCET. (2018) 391:2537–45. doi: 10.1016/S0140-6736(18)31078-X 29976470

[22] Li B Wang C He W Qiu J Zheng Y Zou R. Watch-and-wait strategy vs. resection in patients with radiologic complete response after conversion therapy for initially unresectable hepatocellular carcinoma: a propensity score-matching comparative study. Int J OF SURGERY. (2024) 110:2545–55. doi: 10.1097/JS9.0000000000001155 PMC11093428 38329081

[23] Stahl M Stuschke M Lehmann N Meyer HJ Walz MK Seeber S. Chemoradiation with and without surgery in patients with locally advanced squamous cell carcinoma of the esophagus. J Clin Oncol. (2005) 23:2310–7. doi: 10.1200/JCO.2005.00.034 15800321

[24] Chidambaram S Owen R Sgromo B Chmura M Kisiel A Evans R. Delayed surgical intervention after chemoradiotherapy in esophageal cancer: (DICE) study. Ann Surg. (2023) 278:701–8. doi: 10.1097/SLA.0000000000006028 37477039

[25] Noordman BJ Spaander MCW Valkema R Wijnhoven BPL van Berge HMI Shapiro J. Detection of residual disease after neoadjuvant chemoradiotherapy for oesophageal cancer (preSANO): a prospective multicentre, diagnostic cohort study. Lancet Oncol. (2018) 19:965–74. doi: 10.1016/S1470-2045(18)30201-8 29861116

[26] van der Wilk BJ Eyck BM Hofstetter WL Ajani JA Piessen G Castoro C. Chemoradiotherapy followed by active surveillance versus standard esophagectomy for esophageal cancer: A systematic review and individual patient data meta-analysis. Ann Surg. (2022) 275:467–76. doi: 10.1097/SLA.0000000000004930 34191461

[27] Kelly RJ Ajani JA Kuzdzal J Zander T Van Cutsem E Piessen G. Adjuvant nivolumab in resected esophageal or gastroesophageal junction cancer. N Engl J Med. (2021) 384:1191–203. doi: 10.1056/NEJMoa2032125 33789008

[28] Felip E Altorki N Zhou C Csoszi T Vynnychenko I Goloborodko O. Adjuvant atezolizumab after adjuvant chemotherapy in resected stage IB-IIIA non-small-cell lung cancer (IMpower010): a randomised, multicentre, open-label, phase 3 trial. Lancet. (2021) 398:1344–57. doi: 10.1016/S0140-6736(21)02098-5 34555333

[29] O'Brien M Paz-Ares L Marreaud S Dafni U Oselin K Havel L. Pembrolizumab versus placebo as adjuvant therapy for completely resected stage IB-IIIA non-small-cell lung cancer (PEARLS/KEYNOTE-091): an interim analysis of a randomised, triple-blind, phase 3 trial. Lancet Oncol. (2022) 23:1274–86. doi: 10.1016/S1470-2045(22)00518-6 36108662

[30] Powles T Tomczak P Park SH Venugopal B Ferguson T Symeonides SN. Pembrolizumab versus placebo as post-nephrectomy adjuvant therapy for clear cell renal cell carcinoma (KEYNOTE-564): 30-month follow-up analysis of a multicentre, randomised, double-blind, placebo-controlled, phase 3 trial. Lancet Oncol. (2022) 23:1133–44. doi: 10.1016/S1470-2045(22)00487-9 36055304

[31] Provencio M Nadal E Gonzalez-Larriba JL Martinez-Marti A Bernabe R Bosch-Barrera J. Perioperative nivolumab and chemotherapy in stage III non-small-cell lung cancer. N Engl J Med. (2023) 389:504–13. doi: 10.1056/NEJMoa2215530 37379158

[32] Heymach JV Harpole D Mitsudomi T Taube JM Galffy G Hochmair M. Perioperative durvalumab for resectable non-small-cell lung cancer. N Engl J Med. (2023) 389:1672–84. doi: 10.1056/NEJMoa2304875 37870974

[33] Wang J Qin J Jing S Liu Q Cheng Y Wang Y. Clinical complete response after chemoradiotherapy for carcinoma of thoracic esophagus: Is esophagectomy always necessary? A systematic review and meta-analysis. Thorac Cancer. (2018) 9:1638–47. doi: 10.1111/tca.2018.9.issue-12 PMC6275815 30277016

[34] Waterhouse DM Garon EB Chandler J McCleod M Hussein M Jotte R. Continuous versus 1-year fixed-duration nivolumab in previously treated advanced non-small-cell lung cancer: checkMate 153. J Clin Oncol. (2020) 38:3863–73. doi: 10.1200/JCO.20.00131 PMC7676888 32910710

[35] Antonia SJ Borghaei H Ramalingam SS Horn L De Castro CJ Pluzanski A. Four-year survival with nivolumab in patients with previously treated advanced non-small-cell lung cancer: a pooled analysis. Lancet Oncol. (2019) 20:1395–408. doi: 10.1016/S1470-2045(19)30407-3 PMC7193685 31422028

[36] Yang H Liu H Chen Y Zhu C Fang W Yu Z. Neoadjuvant chemoradiotherapy followed by surgery versus surgery alone for locally advanced squamous cell carcinoma of the esophagus (NEOCRTEC5010): A phase III multicenter, randomized, open-label clinical trial. J Clin Oncol. (2018) 36:2796–803. doi: 10.1200/JCO.2018.79.1483 PMC6145832 30089078

[37] Shapiro J van Lanschot JJB Hulshof MCCM van Hagen P van Berge Henegouwen MI Wijnhoven BPL. Neoadjuvant chemoradiotherapy plus surgery versus surgery alone for oesophageal or junctional cancer (CROSS): long-term results of a randomised controlled trial. Lancet Oncol. (2015) 16:1090–8. doi: 10.1016/S1470-2045(15)00040-6 26254683

[38] Kato K Ito Y Daiko H Ozawa S Ogata T Hara H. A randomized controlled phase III trial comparing two chemotherapy regimen and chemoradiotherapy regimen as neoadjuvant treatment for locally advanced esophageal cancer, JCOG1109 NExT study. J Clin Oncol. (2022) 40:238. doi: 10.1200/JCO.2022.40.4_suppl.238

[39] Jeong Y Kim JH Kim SB Yoon DH Park SI Kim YH. Role of surgical resection in complete responders on FDG-PET after chemoradiotherapy for locally advanced esophageal squamous cell carcinoma. J Surg Oncol. (2014) 109:472–7. doi: 10.1002/jso.v109.5 24301552

[40] Chao YK Tseng CK Wen YW Liu YH Wan YL Chiu CT. Using pretreatment tumor depth and length to select esophageal squamous cell carcinoma patients for nonoperative treatment after neoadjuvant chemoradiotherapy. Ann Surg Oncol. (2013) 20:3000–8. doi: 10.1245/s10434-013-2962-1 23584515

[41] de Gouw D Klarenbeek BR Driessen M Bouwense SAW van Workum F Futterer JJ. Detecting pathological complete response in esophageal cancer after neoadjuvant therapy based on imaging techniques: A diagnostic systematic review and meta-analysis. J Thorac Oncol. (2019) 14:1156–71. doi: 10.1016/j.jtho.2019.04.004 30999111

[42] Pan Y Zhang J-T Gao X Chen Z-Y Yan B Tan P-X. Dynamic circulating tumor DNA during chemoradiotherapy predicts clinical outcomes for locally advanced non-small cell lung cancer patients. Cancer Cell. (2023) 41:1763–+. doi: 10.1016/j.ccell.2023.09.007 37816331

[43] Assaf ZJF Zou W Fine AD Socinski MA Young A Lipson D. A longitudinal circulating tumor DNA-based model associated with survival in metastatic non-small-cell lung cancer. Nat Med. (2023) 29:859–868. doi: 10.1038/s41591-023-02226-6 36928816 PMC10115641

[44] Xu L Wei XF Li CJ Yang ZY Yu YK Li HM. Pathologic responses and surgical outcomes after neoadjuvant immunochemotherapy versus neoadjuvant chemoradiotherapy in patients with locally advanced esophageal squamous cell carcinoma. Front Immunol. (2022) 13:1052542. doi: 10.3389/fimmu.2022.1052542 36466925 PMC9713810

[45] Tang H Jiang D Zhang S Zeng Z Tan L Hou Y. Residual tumor characteristics of esophageal squamous cell carcinoma after neoadjuvant chemoradiotherapy. J Thorac Cardiovasc Surg. (2021) 162:1632–41. doi: 10.1016/j.jtcvs.2020.09.042 33268125

